# Colonic lipoma, a rare cause of intestinal intussusception: A narrative review and how to diagnose it

**DOI:** 10.1097/MD.0000000000039579

**Published:** 2024-09-27

**Authors:** Michele Fiordaliso, Urbano M. Lovaglio, Flavia Antonia De Marco, Raffaele Costantini, Gennaro A. Nasti, Pierluigi Lelli Chiesa

**Affiliations:** a Department of Medicine and Ageing Sciences, University “G. D’Annunzio” of Chieti-Pescara, Pescara, Italy; b Department of Primary Care, A.S.P. Potenza, Potenza, Italy; c Department of Medicine and Gastroenterology, Friedrichshafen, Germany; d Department of Medical, Institute of Surgical Pathology, Oral and Biotechnological Sciences, “G. d’Annunzio” University of Chieti, Pescara, Italy; e Department of General Surgery, Lagonegro-San Carlo Hospital, Potenza, Italy; f Pediatric Surgery Unit, Hospital “Santo Spirito” of Pescara and University “G. d’Annunzio” of Chieti Pescara, Pescara, Italy.

**Keywords:** colonic lipoma, colonic lipoma symptoms, intestinal intussusception, radiology and treatment

## Abstract

Colonic lipomas (CLs) are benign tumors of the adipose tissue of the gastrointestinal tract that are often asymptomatic. A search of medical literature in English using PubMed and Google Scholar was conducted for articles related to CL. Occasionally, patients present with intestinal bleeding or obstructive symptoms. Although intussusception is commonly observed in children, it is rare in adults. Moreover, CL as the most common entity, is very rare, with an incidence rate of 0.035% to 4.4%. Although fatty composition can assist in diagnosis through computed tomography and magnetic resonance imaging, the latter cannot exclude local infiltration. CLs are distributed evenly between both sexes and can be located anywhere in the gastrointestinal tract; however, they are more frequently located in the colon, particularly in the right colon and cecum (39.6%), followed by the transverse colon (25%), descending colon (20.8%), and the sigmoid colon (14.6%). Symptoms included abdominal pain (79.2%), alterations in bowel habits (45.8%), rectal bleeding (22.9%), colocolic intussusception (50%), weight loss (6.2%), vomiting (14.6%), and nausea (12.5%). Surgical and endoscopic techniques are widely used to manage CLs. The challenge for physicians is differentiating this lesion from malignant colonic lesions, at the outset. The risk of misdiagnosis is possible, and the recommendation in cases of doubt is still segmental surgical resection, as it ensures correct collection of lymph nodes for appropriate staging of presumed colonic carcinoma.

## 1. Introduction

Bauer first described colonic lipomas (CLs) in 1757.^[1]^ The prevalence of this lesion ranges from 0.2% to 4.4%, representing 1.8% of all benign colonic lesions,^[2]^ after hyperplastic and adenomatous polyps. Many authors have described a slight predominance of this lesion in females in their 5th or 6th decade of life,^[3]^ primarily localized to the right colon.^[4]^ The lesion is usually segmental, although there have been cases of diffuse lesions reported in the literature, which are typically small and asymptomatic.^[5]^ Therefore, in most cases, they are discovered incidentally. Multiple lipomas have been shown in 10% to 20% of patients, notably if a lipoma was found in the cecum. The challenge for every physician is to differentiate this lesion from malignant colonic lesions, at the outset. Various diagnostic methods are available, including ultrasound, computed tomography (CT), magnetic resonance imaging (MRI), and endoscopic ultrasonography (EUS). In this review, we provide the necessary elements to preoperatively suspect this lesion. Both surgical and endoscopic resections (ERs) have good outcomes with no known recurrence after complete removal. Endoscopy is an effective diagnostic and therapeutic tool for treating small lesions. Surgery is the only method available for large lesions or those involving the muscular or serosal layers. Colotomy excision or segmental colon resection is indicated when the diagnosis is definite. In contrast, segmental resection, hemicolectomy, or subtotal colectomy are recommended when the diagnosis is questionable or when a complication occurs. Indeed, only a histopathological analysis of the tumor can provide a definitive diagnosis. However, this lesion sometimes presents as an acute abdominal condition owing to the occurrence of intussusception with intestinal obstruction. In such cases, time is limited and not all means are available to direct the diagnosis towards the suspicion of a lipoma; therefore, in these cases, it is advisable to consider this tumor as malignant.

## 2. Methods

A search of medical literature in English using PubMed and Google Scholar was conducted for articles related to CL. Search words were: CL, colon lipoma, intussusception, CL symptoms, radiology and treatment. If data were missing, the corresponding authors of the articles in question were contacted by email. Articles containing adequate information, such as publication year, patient age, sex, symptoms, radiological tools, dimensions of the lipoma, and surgical or endoscopic approach were included, while studies and comment articles with insufficient clinical and demographic data and patients overlapping with others were excluded. For duplicate publications, the latest and most complete study was included. Information regarding the demographic characteristics of the study subjects, patient symptoms and physical examinations, operative methods, dimensions and position of the lipoma were extracted from each study report and tabulated in Microsoft Excel. All articles selected for full-text review were examined by the reviewer who decided on inclusion or exclusion and the extraction of the study data.

## 3. Results

The clinicopathological features of symptomatic lipomas have been reviewed in the literature and are summarized in Table 1. In this review, it was found that CLs tended to be distributed in equal proportions between both sexes. The most common ages were in the fifth or sixth decades. Lipomas were in all locations of the gastrointestinal (GI) tract, from the hypopharynx to the rectum, but they were more frequently located in the colon, particularly in the right colon and cecum (39.6%), followed by the transverse colon (25%), descending colon (20.8%), and sigmoid colon (14.6%). Lipomas are usually asymptomatic, particularly when they are smaller than 2 cm in diameter, and are therefore discovered incidentally. Symptoms^[15,16–37]^ were always present for lipomas larger than 4 cm in diameter: abdominal pain (79.2%), alteration in bowel habits (48.5%), rectal bleeding (22.9%), colocolic intussusception (50%), weight loss (6.2%), vomiting (14.6%), and nausea (12.5%). For lipomas causing an intussusception, resulting in intestinal obstruction, the most suitable therapy is surgery. Surgery, which was laparoscopic or open, included: right colectomy (41.7%), segmental colectomy (33.3%), and left colectomy (10.4%).

**Table 1:** Preoperative symptoms and treatment of 41 cases of colonic lipoma.

	Sex		Age	Sign and symptoms						Location				MD	Intussusception	Treatment						
	M	F		AP	BL	AIBH	NA	VO	WL	AC	TC	DC	SC			Laparotomy			Laparoscopy			ER
																RH	LH	SR	RH	LH	SR	
Ref.^[1]^		1	53	1						1				5	YES	1						
Ref.^[3]^	1		42	1		1			1			1		8.5	NO		1					
Ref.^[6]^		1	83							1				3.5	NO	1						
Ref.^[7]^	1		29	1						1				12.5	YES	1						
Ref.^[8]^		1	51	1							1			4.5	YES	1						
Ref.^[9]^		1	54	1								1		6	YES		1					
Ref.^[10]^	1		40	1	1							1		6	YES			1				
Ref.^[11]^		1	27			1							1	2.5	NO						1	
Ref.^[12]^		1	55	1	1	1					1			12	YES		1					
Ref.^[13]^	1		60			1					1			5	NO							1
Ref.^[14]^		1	45	1		1				1				5	YES			1				
Ref.^[15]^		1	56	1			1	1			1			5	NO	1						
Ref.^[5]^		1	67	1							1			5	NO			1				
Ref.^[16]^		1	57	1	1		1	1				1		6	YES		1					
Ref.^[17]^	1		57	1				1				1		6	YES						1	
Ref.^[18]^		1	47			1						1		13	YES						1	
Ref.^[19]^		1	56	1					1		1			8.5	NO				1			
Ref.^[20]^		1	34	1						1				6	YES	1						
Ref.^[21]^		1	49	1		1					1			9	NO				1			
Ref.^[21]^	1		65		1	1							1	3.5	YES						1	
Ref.^[22]^	1		61						1	1				4	YES	1						
Ref.^[23]^	1		67										1	3	NO							1
Ref.^[24]^	1		72								1			4	NO							1
Ref.^[25]^	1		53				1	1		1				4	NO						1	
Ref.^[25]^		1	57	1	1						1			4	NO						1	
Ref.^[26]^		1	72	1								1		3	NO							1
Ref.^[27]^	1		75	1		1							1	5	NO						1	
Ref.^[28]^	1		41	1		1						1		5	NO							1
Ref.^[28]^	1		48	1						1				7	NO							1
Ref.^[29]^	1		49	1	1	1							1	5	YES						1	
Ref.^[30]^	1		50		1					1				3.5	YES	1						
Ref.^[31]^		1	51	1							1			7	YES			1				
Ref.^[32]^		1	51	1		1				1				7	YES	1						
Ref.^[33]^	1		56	1		1				1				6	YES				1			
Ref.^[34]^	1		52	1	1	1						1		5.6	YES					1		
Ref.^[34]^	1		58	1	1								1	3.5	NO						1	
Ref.^[35]^	1		82	1	1	1							1	8	YES							1
Ref.^[36]^	1		45	1				1		1				4	YES	1						
Ref.^[36]^	1		60	1			1	1		1				6	YES	1						
Ref.^[36]^		1	40	1		1				1				6	YES	1						
Ref.^[37]^		1	55	1		1						1		5	YES						1	
Ref.^[38]^		1	69	1	1					1				8	NO	1						
Ref.^[38]^		1	63	1		1				1				7	NO	1						
Ref.^[38]^	1		64	1						1				9	NO	1						
Ref.^[38]^		1	55	1		1					1			7	NO						1	
Ref.^[38]^	1		48	1		1	1			1				6	NO				1			
Ref.^[38]^		1	53	1		1				1				7	NO				1			
Ref.^[38]^	1		45	1		1	1	1			1			8	NO						1	
**Total**	**24**	**24**	**MA = 54.6**	**38**	**11**	**22**	**6**	**7**	**3**	**19**	**12**	**10**	**7**	**MMD = 6.2**	**YES = 24; NO = 24**	**15**	**4**	**4**	**5**	**1**	**12**	**7**
**%**	**50**	**50**	**/**	**79.2**	**22.9**	**45.8**	**12.5**	**14.6**	**6.2**	**39.6**	**25**	**20.8**	**14.6**	**/**	**YES = 50%**	**31.3**	**8.3**	**8.3**	**10.4**	**2.1**	**25**	**14.6**

AC = ascending colon, AIBH = alteration in bowel habits, AP = abdominal pain, BL = bleeding, DC = descending colon, ER = endoscopical remove, LH = left hemicolectomy, NA = nausea, RH = right hemicolectomy, SC = sigmoid colon, SR = segmental resection, TC = transverse colon, VO = vomit, WL = weight loss.

## 4. Discussion

**Figure 1.**
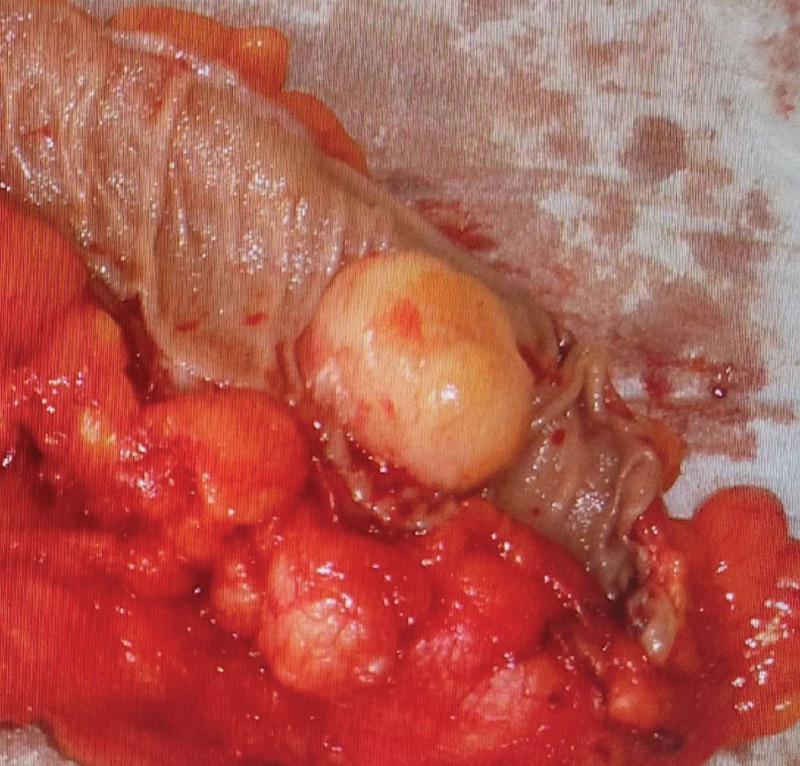
Surgical specimen: submusosal tumor of the descending colon measuring 5 cm.

**Figure 2.**
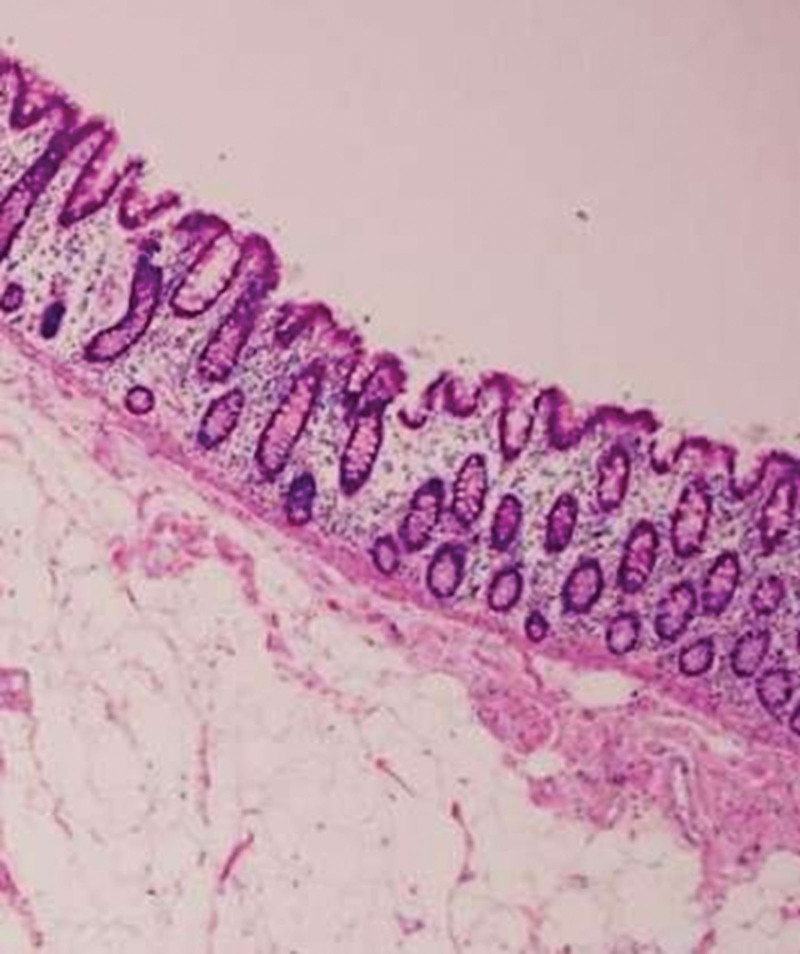
Histology of lesion compatible with submucosal lipoma.

The etiology of CL is unclear. It has been hypothesized that chronic intestinal irritation, inflammatory responses, and adipose tissue accumulation may be responsible for the formation of CLs. Other theories suggest a connection between soft tissue trauma and the formation of lipomas; they are pseudolipomas due to the prolapse of adipose tissue or that they are related to the proliferation of pre-adipocytes activated by circulating cytokines.^[39,40]^ Some genetic anomalies have been found to be responsible for the formation of lipomas, such as mutations in chromosome 12q13-15, deletions of 13q, and rearrangements of 6p21-33.^[41]^ Additionally, intestinal lipomas can be associated with other conditions, such as multiple hereditary lipomatosis, adiposis dolorosa, Gardner syndrome, and Cowden syndrome.^[42,43]^ Macroscopically, lipomas appear yellowish, well circumscribed, soft, and compressible (Fig. 1). Various degrees of necrosis, granulation, and ulceration are observed on the surfaces of large lipomas. Histopathologically, they are mesenchymal tumors composed of well-differentiated adipocytes supported by fibrous tissue, which tends to give the lipoma a lobulated structure, and there is usually a thick capsule surrounding the tumor (Fig. 2). Larger lesions can become lobulated or pedunculated. They generally originate in the submucosa^[17]^ but occasionally extend to the muscularis propria^[30]^; 10% are subserosal,^[44]^ whereas transmural localization is extremely rare. Secondary cellular changes include nuclear hypertrophy, hyperchromasia, pleomorphism, and fat necrosis. Malignant transformation has never been reported, although some lipomas may exhibit atypical histological characteristics described as “pseudosarcomatous”^[45–47]^; however, frank liposarcoma of the colon is extremely rare and requires the presence of lipoblasts. Recurrence after surgical intervention has not been reported. However, in 25% of cases, patients present with obstructive symptoms, not so much due to the lipoma, as these are mostly small, but due to intestinal intussusception induced by the lipoma.^[48]^ We demonstrated a significant correlation between tumor size and intussusception. The larger the tumor, the higher the likelihood that the patient would present with intussusception. Intussusception occurs when a more proximal portion of the bowel (intussusceptum) invaginates into the more distal bowel (intussuscipiens).^[5,14–23]^ The pathological mechanism is thought to involve altered bowel peristalsis at the intraluminal lesion, which is a lead point for the intussusceptum. Although intussusception is common in children, it is rare in adults.^[7,8]^ The latter differs from pediatric intussusception in various aspects, including etiology and clinical characteristics. In contrast to intussusceptions in children, in adults, a demonstrable etiology is found in 70% to 95% of cases, and primary or secondary malignant tumors are the cause of approximately 40% of intussusceptions. The tumors causing intussusception of the colon include adenocarcinoma, metastatic carcinoma, primary adenocarcinoma, GI stromal tumors, lymphoma, carcinoid tumors, and metastatic malignant neoplasms with intestinal localization. Benign tumors that cause intussusception are leiomyomas, adenomas, lipomas, Brunner cell hamartomas, hemangiomas, adenomyomas, neurofibromas, and desmoid tumors. The cause of intussusception is unknown in 90% of pediatric cases.^[49]^ There is no recognized etiology of the abnormal peristaltic wave that leads to the invagination. This leads to compression and angulation of the mesenteric vessels, resulting in reduced perfusion, venous congestion, edema of the intestinal wall, ischemia, and possible intestinal necrosis with bowel perforation. Many patients with intussusception present with various combinations of nonspecific symptoms, including nausea, vomiting, and abdominal pain, and it may initially be confused with other abdominal pathologies; differentiation from other types of visceral pain plays an important role.^[49,50]^ Intussusceptions are classified into 4 categories according to the location in the intestinal tract: enteric, ileocolic, ileocecal, or colic.^[51–53]^ The enteric^[52]^ and colic forms involve the small (jejunum or ileum) and large intestines, respectively. One remaining challenge in the management of CLs is establishing a preoperative diagnosis. Sonography is one of the first available, rapid, and noninvasive examinations for clinicians. CLs are characterized by a solid hyperechoic mass with or without minimal vascularity.^[54]^ However, the size of the mass, a patient with a high body mass index, and intestinal gas can make the diagnosis, even in the hands of an expert operator, difficult to differentiate from other lesions originating from the intestinal wall or migrating from other sites,^[55,56]^ as could be the case with a large gallstone migrating to the intestine.^[56]^ Gallstones are notoriously difficult to visualize on plain film radiography, with only 10% to 20% of stones containing sufficient calcium to be radio-opaque.^[57]^ Of those stones that were not visualized, the vast majority were radiolucent (typically associated with cholesterol stones). In this case, CT with intravenous contrast can assist in differentiating between the 2 lesions. Furthermore, the drawback of intravenous contrast-enhanced CT imaging for gallstone ileus is the difficulty in defining some radiolucent stones or rim-calcified stones. Intraluminal radiolucent stones may resemble soft tissue densities (iso-attenuation), such as a mass or an intussusceptum.^[57]^ Another method to diagnose CLs, often used in the past, is the barium enema test (27%), which has a dual diagnostic and therapeutic capacity, particularly for patients who present with an intussusception.^[47]^ A radiological pathognomonic sign of a CL is the squeeze sign, in which a radiolucent filling defect with visible margins is shown to change in size and shape owing to peristaltic activity when a barium enema is administered. However, the diagnostic sensitivity is approximately 36%, making it difficult to differentiate a colon lipoma from any other type of colon tumor; therefore, its use remains debatable. This examination was widely used before the 2000s; however, it is currently less utilized and has been replaced by CT^[21–29]^ and EUS. For patients with typical features of CLs, CT reliably confirms the diagnosis. A lipoma has a uniform appearance on a CT image with fat-equivalent density and a smooth border (low-attenuation lesion: ‐40 to ‐120 Hounsfield units). The sensitivity of CT imaging to diagnose intussusception is 71.4% to 87.5%, and its specificity is 100%, in adults. However, intussuscepted lipomas may not demonstrate normal fat attenuation and may have a heterogeneous appearance, reflecting the degree of infarction and fat necrosis present at the time of radiological evaluation, leading to a diagnostic dilemma. On CT images, it is possible to identify a well-defined lesion with regular margins and fatty density^[58]^ but it is not possible to differentiate a benign lesion from a malignant lesion. However, the diagnosis of small lipomas can be difficult using CT. In addition to CT, MRI can provide indications for diagnosis, particularly in patients with large colon lipomas. MRI is highly effective in highlighting adipose lesions because of the peculiar characteristics of the signal intensity of this tissue, particularly in T1-weighted and fat-suppressed images. However, this imaging modality is rarely used to detect or study intestinal neoplastic lesions.^[59]^ EUS is another modality that can be used to differentiate lipomas of the colon from other pathologies. The GI wall is detected as a five-layer structure with a lower frequency (7.5–12 MHz) and a nine-layer structure with a higher frequency (12–20 MHz). EUS is useful for characterizing submucosal tumors because it identifies the layer of origin of the submucosal lesion and the internal echo pattern. Lipomas, lymphangiomas, and fibromas originate from the third layer. With regard to the echo level of subepithelial lesions, because lipomas have a characteristic hyperechoic pattern, it is not necessary to perform EUS fine-needle aspiration. Furthermore, EUS minimizes the complications associated with endoscopic removal. Spontaneous resolution of GI lipoma is rare but the literature describes the resolution of a gastric lipoma after endoscopic biopsy. There are several possible explanations for the phenomenon. The most accepted explanation is that a biopsy causes a lesion in the mucous layer immediately above the lipoma, which would initially favor the exposure of the tumor and subsequent enucleation. Some cases of resolution of lipomas are described in the presence of pedunculated lipomas, either spontaneously or after biopsy. In addition to the hypothesis previously described for mucosal lesions, another explanation could be linked to a twist of the pedicle with ischemia and subsequent detachment of the lipoma. Through colonoscopy, it is possible to not only visualize the lesion but to also perform a biopsy. Macroscopically, the lesion appears smooth, spheroidal, yellowish, of varying sizes, with or without a pedunculated base. Three endoscopic signs can direct clinical suspicion towards the diagnosis of lipoma: “cushion sign”^[59]^ (probing the polyp with closed biopsy forceps will often yield a pillow-like indentation), “tenting effect”^[59]^ (grasping the overlying mucosa with the biopsy forceps presents a tent-like appearance), and the “naked fat sign”^[60]^ where yellowish fat can be extruded after biopsy. A biopsy is not always helpful because the histological presence of fat cannot exclude the concurrent presence of neoplastic cells within the lesion. Only complete histopathological analysis of the specimen can provide a definitive diagnosis. Treatment can be performed endoscopically, laparoscopically,^[25–33]^ or by using an open technique. There are conflicting opinions in the literature regarding the maximum dimensions of the lesion for which a safe ER can be performed. Many authors report that a 2 cm in diameter lipoma is the maximum limit to avoid risking perforation.^[6]^ Bar-Meir et al^[61]^ have described safe endoscopic removal of a very large 5-cm in diameter lipoma.^[9–12]^ However, pedunculated lipomas up to 11 cm in diameter have been safely removed endoscopically using newer techniques, such as snare electrosurgery or endoloop ligation. ER should be avoided in patients with acute conditions due to a lack of bowel preparation, and in cases of intussusception due to the possible ischemia of the affected segment, with an increased risk of perforation. Application of endoscopic procedures is limited to tumors arising from the submucosa, and the risk of perforation increases exponentially when deeper layers are involved. Location also plays an important role in the choice of procedure, as the right colon is more susceptible to perforation than the left colon. However, perforation of the cecum can be more easily repaired endoscopically because of the greater maneuverability of the instrument in the much larger intestinal lumen than in the left colon. With the endoscopic technique, several procedures are possible such as the “unroofing” dissection of the lipoma with a loop assisted snare, the “Ligate and Let Go” technique, endoscopic mucosal resection (EMR), and endoscopic submucosal dissection (ESD), all with excellent remission rates.^[62,63]^ EMR is a minimally invasive procedure for removing GI cancers, precancerous tumors, and benign tumors. After identifying the tumor, it is lifted with a submucosal fluid injection (the “inject-and-cut” technique). Lifting facilitates grasping with a polypectomy snare, and by separating the 2 layers, the muscle layer (muscularis propria) is prevented from being included in the resection. The lesions are removed with the assistance of an electrical snare; a thin wire fed through the endoscope, forming a loop at the end that tightens around the tumor. A high-frequency electric current (35 W) crosses the wire, which is applied to remove the lesion and to cauterize the wound. After the lesion is removed, hemorrhage is controlled using electrocoagulation forceps. Endoclips are then used to close the wound site and the resected specimen is sent for histopathological examination. A possible risk that can be incurred with EMR is perforation or delayed perforation and incomplete resection of the lipoma. To avoid these complications, an endoloop before the procedure or hemoclipping after procedure, are recommended. To avoid deep-tissue damage, a blended or pure cut is recommended to avoid excessive coagulation. EMR is most effective for tumors no larger than two 2 cm in diameter and when these do not reach the deep layers of the intestinal wall. Larger lesions must be removed piecemeal. Furthermore, the area to be resected is well delimited from the surrounding tissue, it may be not be possible to remove some larger and deeper tumors in 1 piece using EMR. This makes it difficult to confirm whether all tumor cells have been removed, favoring the development of recurrence. Tumors that reached deeper layers may be treated with ESD or endoscopic full-thickness resection. ESD is a procedure that is poorly developed in Western countries because of technical difficulties and the considerable experience required. Before ESD, EUS was performed to determine the tumor size, layer of origin, and growth pattern. ESD includes the following steps: identifying and marking tumor boundaries, injecting saline into the submucosal layer, incising the mucosal layer with a hook knife, and dissecting the tumor. During ESD of CLs, electrical energy is concentrated only between the knife and the tissue, and the electrical energy is applied to the submucosal layer to minimize thermal injury. In addition, ESD has the advantage of enabling removal of larger lesions compared to EMR. It has been demonstrated that for lesions below 3 cm in diameter, ER has more advantages than surgical resection with less trauma, early healing, and low cost.^[13–15,16–38]^ If the lesion is too large to be resected endoscopically, surgery is the only alternative. Preoperative tests include blood cell counts, hepatic and renal biochemical functional profiling, and evaluation of cancer markers. Adequate preoperative patient preparation is crucial for positive surgical outcomes. Solid bowel preparation with laxatives, preoperative administration of antibiotics, and prophylactic administration of low-molecular-weight heparin to prevent deep vein thrombosis are routine practices. Colotomy with lipectomy is the preferred treatment in the absence of complications.^[1,40]^ In cases of diagnostic doubt or acute intussusception, segmental colic resection must be considered.^[64,65,66]^ Surgical resection is recommended in the event of colonic intussusception, particularly in older patients, because of the high possibility of a malignant neoplasm. In cases of doubt, an intraoperative frozen section histopathological examination can be helpful. To ensure surgical margin clearance and to limit the excision of the tumor to a confined area, intraoperative frozen section is required, as it can eliminate the need for a more radical procedure, such as hemicolectomy. In doubtful cases or in cases where it was not possible to perform a preoperative diagnosis due to an acute presentation, a hemicolectomy with D2 lymphadenectomy is recommended. Surgical resection should be performed in the following cases: (1) lipomas larger than 4 cm in diameter, (2) preoperative diagnosis suspicious of malignancy, (3) symptomatic lesions or lesions causing intussusception, (4) wall infiltration, and (5) lipomas that cannot be completely removed endoscopically. From this review, despite the various means available to diagnose colon lipomas, it is often not possible to differentiate from carcinomas; in these case, the most suitable approach is removal of the tumor without reduction. Normally, in adults, surgical reduction of the intestinal invagination before resection is not recommended owing to the risk of intestinal perforation. Furthermore, if the intussusception is associated with a malignant tumor, reduction may cause dissemination of tumor cells.^[51]^ If a diagnosis of lipoma has already been made and the patient presents with acute onset, it is advisable to reduce the affected segment to avoid excessive bowel resection. Surgical reduction involves a combination of delicate, direct pressure on the anal aspect of the bowel (intussuscipiens) and gentle pulling on the oral aspect (intussusceptum). Reduction can be performed using both open and laparoscopic techniques. In cases of idiopathic intussusception, particularly in children, resolution similar to endoscopic maneuvers through air insufflation have been described. In the absence of diagnostic doubt, the most reasonable approach for an asymptomatic lipoma is therapeutic abstention.

## 5. Conclusion

Although rare, CLs should be considered in the differential diagnosis of large intestine tumors. Small lipomas are typically asymptomatic and, in such cases, no therapy is required. When they increase in size, they can present with symptoms, such as abdominal pain, hemorrhage, vomiting, and intestinal obstruction. As this is a benign condition, the best approach should be the least destructive to the involved organ, in this case, the colon. For lesions measuring approximately 2 cm in diameter, endoscopic removal by EMR or ESD are the procedures of choice, although ERs of lipomas 4 cm in diameter have been described in the literature, without complications. If endoscopic techniques fail, the only alternative is surgery which can be performed using either open or laparoscopic techniques.^[11]^ A recent publication indicated that laparoscopic resection is a good alternative to open conventional surgery with all the known advantages of minimally invasive procedures, such as reduced postoperative pain, early resumption of daily activities, and early discharge. Colotomy with lipectomy or segmental resection is the procedure of choice because they are less destructive. CLs can also lead to intussusception. When a patient presents with acute intestinal obstruction, it is not always possible to use all currently available diagnostic tools, such as MRI and endoscopy, to differentiate benign from malignant lesions. Surgical treatment includes resection, colotomy with local excision, limited colon resection, segmental resection, hemicolectomy, or subtotal colectomy. Differentiating a benign lipoma from a malignant process before surgery is useful because the diagnosis affects the extent of surgical resection; however, this may not always be possible. Our advice, in doubtful cases, is to consider, particularly in older patients without a previous colonoscopy, that the condition is malignant, and to adhere to the principles of oncological surgery. Surgical reduction followed by colonic resection is an excellent treatment modality for intussusception secondary to CL.

## Author contributions

**Conceptualization:** Michele Fiordaliso.

**Data curation:** Michele Fiordaliso, Urbano M. Lovaglio, Flavia Antonia De Marco, Gennaro A. Nasti, Pierluigi Lelli Chiesa.

**Supervision:** Raffaele Costantini, Pierluigi Lelli Chiesa.

**Writing – original draft:** Michele Fiordaliso, Flavia Antonia De Marco.

**Writing – review & editing:** Michele Fiordaliso, Urbano M. Lovaglio, Raffaele Costantini, Gennaro A. Nasti.
